# Function and mechanism of MCM8 in the development and progression of colorectal cancer

**DOI:** 10.1186/s12967-023-04084-9

**Published:** 2023-09-14

**Authors:** Shaojun Yu, Weixing Dai, Senlin Zhao, Yongzhi Yang, Ye Xu, Jianwei Wang, Qun Deng, Jinghu He, Debing Shi

**Affiliations:** 1 Key Laboratory of Cancer Prevention and Intervention, The Second Affiliated Hospital, Ministry of Education, Zhejiang University School of Medicine, Hangzhou, Zhejiang China; 2 Department of Colorectal Surgery, Fudan University Shanghai Cancer Center, 270 Dong’an Road, Shanghai, 200032 PR China; 3 Department of Oncology, Shanghai Medical College, Fudan University, Shanghai, 200032 China; 4 Department of Colorectal Surgery and Oncology, Key Laboratory of Cancer Prevention and Intervention, The Second Affiliated Hospital, Ministry of Education, Zhejiang University School of Medicine, Hangzhou, Zhejiang China; 5 Department of General Surgery, Changhai Hospital Affiliated to Navy Medical University, Shanghai, China

**Keywords:** Colorectal cancer, MCM8, CHSY1, Tumor promotor, Molecular mechanism

## Abstract

Colorectal cancer (CRC) has become a global health problem which has almost highest morbidity and mortality in all types of cancers. This study aimed to uncover the biological functions and underlying mechanism of MCM8 in the development and progression of CRC. The expression level of MCM8 was found to be upregulated in CRC tissues and significantly associated with tumor grade and patients’ survival. Knocking down MCM8 expression in CRC cells could restrain cell growth and cell motility while promoting cell apoptosis in vitro, as well as inhibit tumor growth in xenograft mice model. Based on the RNA screening performing on CRC cells with or without MCM8 knockdown and the following IPA analysis, CHSY1 was identified as a potential target of MCM8 in CRC, whose expression was also found to be higher in tumor tissues than in normal tissues. Moreover, it was demonstrated that MCM8 may regulate the expression of CHSY1 through affecting its NEDD4-mediated ubiquitination, both of which synergistically execute tumor promotion effects on CRC. In conclusion, the outcomes of our study showed the first evidence that MCM8 act as a tumor promotor in CRC, and may be a promising therapeutic target of CRC treatment.

**Supplementary Information:**

The online version contains supplementary material available at 10.1186/s12967-023-04084-9.

## Introduction

Currently, colorectal cancer (CRC) has become a global health problem whose morbidity and mortality ranked third and second among all types of malignant tumors, respectively, according to the latest statistical analysis [1, 2]. However, the occult incidence of CRC leads to the fact that most patients are in advanced stage when diagnosed with CRC, which brings many difficulties to the clinical treatment of CRC [3]. Emerging evidence indicated that the occurrence and development of CRC is a complex process, involving multiple factors and stages, in which the over-activation of oncogenes and the inactivation of tumor suppressor genes are important characteristics of CRC development [4, 5]. For example, it has been manifested that CRC development involves the deletion of DNA mismatch repair (dMMR) genes, the high expression of oncogenes including c-Myc, RAS, HER-2, mutation of KRAS, BRAF, CD44 and well known tumor suppressor gene p53 etc. [4, 6]. Accordingly, with the rapid development of molecular biology, molecular targeted therapy has become a novel and highly effective treatment method for CRC in the past decades, which is, however, severely limited by the unclear molecular mechanism of CRC [5, 7]. Therefore, the study of molecular mechanism and the exploration of key regulatory factors related to CRC progression can provide theoretical basis for optimizing tumor treatment, and ultimately improve the prognosis of patients [8–10].

Microchromosome maintenance complex (MCM) is a family of proteins involved in DNA replication, meiosis, homologous recombination and repair, and its members include MCM2-10. MCM family members are very similar in structure, all of them have the helicase domain, zinc finger structure and Walker A / B domain [11]. Among MCM family members, MCM8 and MCM9 are two of the most recently concerned proteins. Unlike other members of MCM protein family, MCM8 protein is only expressed in eukaryotes and Drosophila, and is highly conserved among primates. MCM8 can exist stably in the whole cell cycle, and has DNA helicase activity and ATPase activity [12]. It is an important component of replication initiation complex and participates in the process of homologous recombination and repair. When DNA double strand breaks, MCM8 and MCM9 can form heterodimer complexes, interact with RAD51, promote the recruitment of RAD51 at DNA damage sites, and use homologous chromosome sequence as template for DNA synthesis, which is also an important step of meiosis homologous recombination [13–17]. However, except for the role in DNA repairing and recombination, studies concerning the biological functions of MCM8 are still very limited [18]. As for cancer, a meta-analysis of genome-wide copy number recognized the overexpression of MCM8 in a variety of human malignancies [19]. Moreover, MCM8 has recently been identified as key regulator in the development and progression of several types of human cancers such as gastric cancer [20, 21], osteosarcoma [22], bladder cancer [23], glioma [24] and cholangiocarcinoma [25].

To the best of our knowledge, the role of MCM8 in human CRC progression has never been uncovered and remains unclear. In this study, we comprehensively performed a number of molecular biology research technologies to reveal the expression pattern of MCM8 in colorectal cancer and its effect on the progression of colorectal cancer in vitro or in vivo. Moreover, the underlying mechanism was further explored through the combination of microarray analysis and bioinformatics analysis, and verified by functional rescue experiments.

## Materials and methods

### Cell culture

Human colorectal cancer cell lines Caco-2 and RKO were purchased from the Institute of Biochemistry and Cell Biology of the Chinese Academy of Sciences (Shanghai), and HCT116, DLD-1 and SW480 were purchased from BeNa Technology (Hangzhou). Caco-2 cells were cultured in 80% MEM supplemented with 20% fetal bovine serum (FBS, Invitrogen). HCT116 cells were cultured in 90% RPMI 1640 (GIBCO) supplemented with 10% FBS. RKO cells were cultured in 90% EMEM with 10% FBS. SW480 and DLD-1 were maintained in 90% DMEM-H supplemented with 10% FBS.

### Immunohistochemical (IHC) staining

Human colorectal cancer tissues and corresponding adjacent normal tissues microarray was purchased from Shanghai Outdo Biotech Co., Ltd., which were collected from patients with colorectal cancer, pathological characteristics and informed consent form were collected as well. The experiment was approved by Fudan University Shanghai Cancer Center Institutional Review Board (SCCIRB). For IHC assay, slides were dewaxed, rehydrated, and then blocked with 3% H_2_O_2_. Slides were incubated with antibodies at 4 °C overnight. After washing with PBS, slides were incubated with appropriate horseradish peroxidase (HRP)-conjugated IgG polyclonal antibody for 30 min at room temperature. All slides were stained by DAB and hematoxylin. IHC scoring of specimens were determined based on the sum of the staining intensity and staining extent scores. Antibodies used in IHC assay were showed in Additional file 1:  Table S1.

### Lentiviral vector construction

RNA interference and overexpression of target gene human MCM8 or CHSY1 were designed and carried out in Shanghai Bioscienceres, Co., Ltd. and the sequences were showed in Additional file 1: Table S2. ShRNA sequences were reverse transcript to cDNAs and double stranded DNA were obtained. Linearized double stranded DNA were cloned into the linearized BR-V-108 using Fermentas T4 DNA Ligase. The junction products were transformed into E.coli receptor cells. Clones with target sequence were selected for plasmid extraction using EndoFree Maxi Plasmid Kit and qualified plasmids used for viral packaging. Empty lentiviral vector was used as control.

### Cell infection

HCT116 and RKO cells were seeded in a 6-well +plate with 2 × 10^5^ and 400 µL infective fluid including ENI.S plus Polybrene and 1 × 10^7^ TU/well recombinant lentiviruses were added into each well for cell infection. After 72 h culturing, the fluorescence efficiency was observed using fluorescence microscope (OLYMPUS).

### RT-qPCR

Total RNAs in lentivirus infected HCT116 and RKO cells were extracted with Trizol Reagent. Concentration and quality of total RNA was determined by Nanodrop 2000/2000c spectrophotometer. cDNA was obtained by Promega M-MLV Kit (Promega Corporation). Real-time PCR was performed using SYBR Premix Ex Taq with 12 µL reaction system. Relative quantitative RNA levels were calculated by the method of 2^−∆∆CT^. Primer sequences used in PCR experiments were detailed in Additional file 1: Table S3.

### Western blot

Total proteins lentivirus infected HCT116 and RKO cells were lysed by Lysis Buffer and the concentration was measured using BCA Protein Assay Kit (HyClone-Pierce). PVDF membranes. After blocked by TBST with 5% skim milk, the membranes were incubated with primary antibodies (detailed in Additional file 1: Table S1) at 4 °C overnight. We washed with TBST three times and added HRP-conjugated IgG polyclonal antibodies as the secondary antibody incubated for 2 h at room temperature. ECL plusTM Western blotting system kit from Amersham was used for color developing and target proteins detecting. For co-immunoprecipitation, proteins were immunoprecipitated by anti-CHSY1 or anti-MCM8 antibody, followed by western blot analysis with antibody of ubiquitin, MCM8 and NEDD4.

### Cell proliferation

Cell proliferation of the infected HCT116 and RKO cells were detected using MTT assay. 2000 cells/well were seeded in a 96-well plate and maintained in a cell incubator at 37 °C with 5% CO_2_. MTT assay solution (20 µL, 5 mg/mL; Genview) was added into each well for 4 h reaction, and then 100 µL dimethyl sulfoxide (DMSO) were added. Cell proliferation was revealed by microplate reader (Tecan infinite) at 490 nm.

### Cell apoptosis and cycle assay

Infected HCT116 and RKO cells were harvested and centrifuged (1300 rpm) for 5 min, and then washed with 4 °C pre-cooled D-Hanks. Then cells suspension with 5 × 10^5^ cells using 1×binding buffer was stained by 10 µL Annexin V-APC for 15 min in the dark. FACScan (Millipore) was used to assess the apoptosis rate.

shCHSY1 and shMCM8 + shCHSY1 lentivirus infected RKO cells were cultured in a 6 cm dish for 5 days, and then cells were harvested and washed with PBS. After centrifuged (1200 rpm) for 5 min, cells were washed with 4 °C pre-cooled PBS and fixed by 70% ethanol for 1 h. After centrifuged at 1500 rpm, 1 mL PI staining solution was added for cell cycle distribution detecting using FACSCalibur (BD Biosciences) at 200 ~ 300 Cell/s.

### Colony formation assay

Infected HCT116 and RKO cells were cultured for 5 days and then seeded into 6-well plates with 500 cell/well. After cells were further cultured for 8 days (cell culture medium was changed every three days), cells were fixed with 4% paraformaldehyde, then stained with GIEMSA (DingGuo Biotechnology). The clone (> 50 cells) number was counted.

## Wound-healing assay

Cell migration rates was estimated via wound-healing assay using a 96 wounding replicator (VP scientific). Briefly, shCHSY1 and shMCM8 + shCHSY1 lentivirus infected RKO cells (5 × 10^4^ cells/well) were seeded into a 96-well dish for culturing until cells confluence reached 90%, and scratches across the cell layer were made. The dish was gently rinsed with PBS. Photographs were taken at 20 and 30 h post scratching using a fluorescence microscope.

### Transwell assay

The migration ability of lentivirus infected RKO cells was analyzed by transwell assay using a Corning Transwell Kit. 100 µL medium without FBS was added into the 24-well plate in the upper chamber, 1 h later, medium was removed and 100 µL exponentially growing RKO cells suspension (1 × 10^5^ cells) was seeded into the plate. The lower chamber was filled with 600 µL containing 30% FBS. Cells were cultured for 24 h at 37 °C with 5% CO_2_. Cell medium and non-metastatic cells were removed. Metastatic cells were fixed by 4% formaldehyde and 400 µL Giemsa was added for staining.

### Human apoptosis antibody array

In lentivirus infected RKO cells, human apoptosis antibody array was applied to detecte the related genes in human apoptosis signal pathway. Each membrane was blocked with Blocking Buffer and incubated in cell lysis Buffer overnight at 4 °C. After that, membranes were incubated in Biotin-conjugated Anti-Cytokines overnight at 4 °C. Streptavidin-HRP was added to incubate at room temperature for 2 h. The signals were detected using enhanced chemiluminescence (ECL).

## Genechip microarray

Total RNA was extracted by TRIZOL Reagent (Life technologies) following the manufacturer’s instructions. RNA integrity was qualified by Agilent Bioanalyzer 2100 (Agilent technologies). Affymetrix human GeneChip 3’ IVT PLUS Reagent Kit was used to obtain biotin labeled cRNA according to the manufacturer’s instruction and array hybridization and wash was performed using GeneChip® Hybridization, Wash and Stain Kit (Affymetrix). Slides were scanned by GeneChip® Scanner 3000 and Command Console Software 4.0 with default settings. Raw data statistical significance assessment was accomplished using a Welch t-test with Benjamini-Hochberg FDR (< 0.05 as significant). Significant difference analysis and functional analysis based on Ingenuity Pathway Analysis (IPA) (Qiagen).

### Tumor-bearing mice model

200 µL cell suspension (4 × 10^6^ cells) of lentivirus infected logarithmic growth phase RKO cells was injected subcutaneously into female BALB/c nude mice (4 week-old) (10 for each group), which were purchased from Shanghai Lingchang Laboratory Animal Co. Ltd. Tumors volume and weight of mice was measured 1–2 times per week. Before terminated the experiment, all mice were anesthetized by sodium pentobarbital (0.7%, 10 µL/g) intraperitoneal injection and placed under an IVIS Spectrum (Perkin Elmer) for bioluminescent imaging. Then all mice were sacrificed and the tumors were removed. In addition, Ki-67 expression level in tumor tissues was determined by Ki-67 immunostaining assay. All the animal experiments were approved by Fudan University Shanghai Cancer Center Institutional Review Board (SCCIRB).

### Statistical analysis

Each experiment was carried out 3 times under the same conditions. Data were showed as mean ± standard deviation (SD) for each experimental group. Significant differences between two groups were assessed with Student’s t-test. Differences of multiple groups were analyzed with one-way ANOVA analysis for parametric data or Mann-Whitney U test for non-parametric data via GraphPad Prism 6 (San Diego). *P* < 0.05 was considered statistically significant.

## Results

### MCM8 is abnormally upregulated in CRC tissues and abundantly expressed in CRC cells

**Fig. 1:**
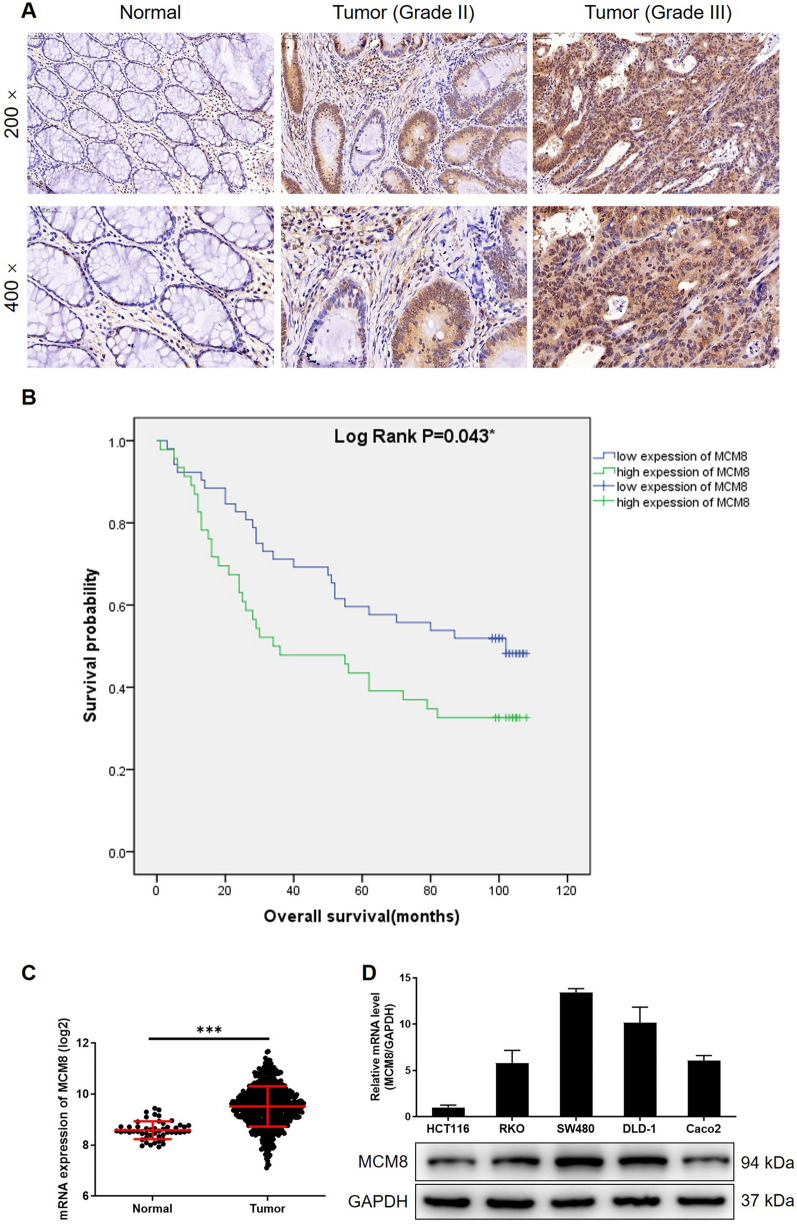
MCM8 is highly expressed in CRC. **A** The expression of MCM8 in normal tissues and CRC tissues with different pathological stage was detected by IHC. **B** The relationship between MCM8 expression and CRC patients’ prognosis was analyzed by Kaplan-Meier survival analysis. **C** The expression (log2 value) profiling of MCM8 in normal tissues and CRC tissues was collected from TCGA database for bioinformatics analysis. **D** The endogenous mRNA and protein expression of MCM8 in CRC cell lines including HCT116, RKO, SW480, DLD-1 and Caco2 was examined by qPCR. Data were shown as mean ± standard deviation (SD). *P < 0.05, **P < 0.01, ***P < 0.001

The evaluation of MCM8 expression in CRC and corresponding normal tissues was performed by IHC to preliminarily explore its potential function in CRC. As shown in Fig. 1A, CRC tissues exhibited obviously higher protein expression of MCM8 relative to normal tissues. Moreover, as judged from the MCM8 expression in tumor tissues with different grade, it is clear that MCM8 expression increases with the severity of tumors (Fig. 1A). The statistical analysis between MCM8 expression in 98 clinical specimens and tumor characteristics also showed that MCM8 was significantly upregulated in CRC and related to tumor grade (Tables 1 and 2 and Additional file 1: Table S4). Moreover, through the Kaplan-Meier survival analysis, high expression of MCM8 may predict shorter survival period of CRC patients (Fig. 1B). Consistently, we also consulted the gene expression profiling in TCGA database, which verified the upregulation of MCM8 in CRC (Fig. 1C). Before conducting in vitro experiments, we found abundant endogenous mRNA and protein expression of MCM8 in all tested CRC cell lines including HCT116, RKO, SW480, DLD-1 and Caco2 (Fig. 1D). Therefore, HCT116 and RKO cells were chosen for subsequent loss-of-function studies in vitro.

**Table 1:** Expression patterns of MCM8 in colorectal cancer tissues and normal tissues revealed in immunohistochemistry analysis

MCM8 expression	Tumor tissue		Normal tissue	
	Cases	Percentage (%)	Cases	Percentage
Low	52	53.1%	74	100%
High	46	46.9%	0	0%

*P* < 0.001

**Table 2:** Relationship between MCM8 expression and tumor characteristics in patients with colorectal cancer

Features	No. of patients	MCM8 expression		*P* value
		low	high	
All patients	98	52	46	
Age (years)				0.665
< 71	44	24	20	
≥ 71	48	24	24	
Gender				0.725
Male	53	27	26	
Female	44	24	20	
Tumor size				0.675
< 5.5 cm	47	26	21	
≥ 5.5 cm	49	25	24	
Grade				0.017
II	51	33	18	
III	47	19	28	
Stage				0.232
1	5	2	3	
2	53	33	20	
3	36	15	21	
4	3	2	1	
T Infiltrate				0.077
T1	1	0	1	
T2	5	3	2	
T3	75	44	31	
T4	13	3	10	
lymphatic metastasis (N)				0.072
N0	58	35	23	
N1	27	12	15	
N2	11	4	7	
Lymph node positive				0.269
= 0	48	26	22	
> 0	38	16	22	

### MCM8 deficiency inhibits development of CRC in vitro

**Fig. 2:**
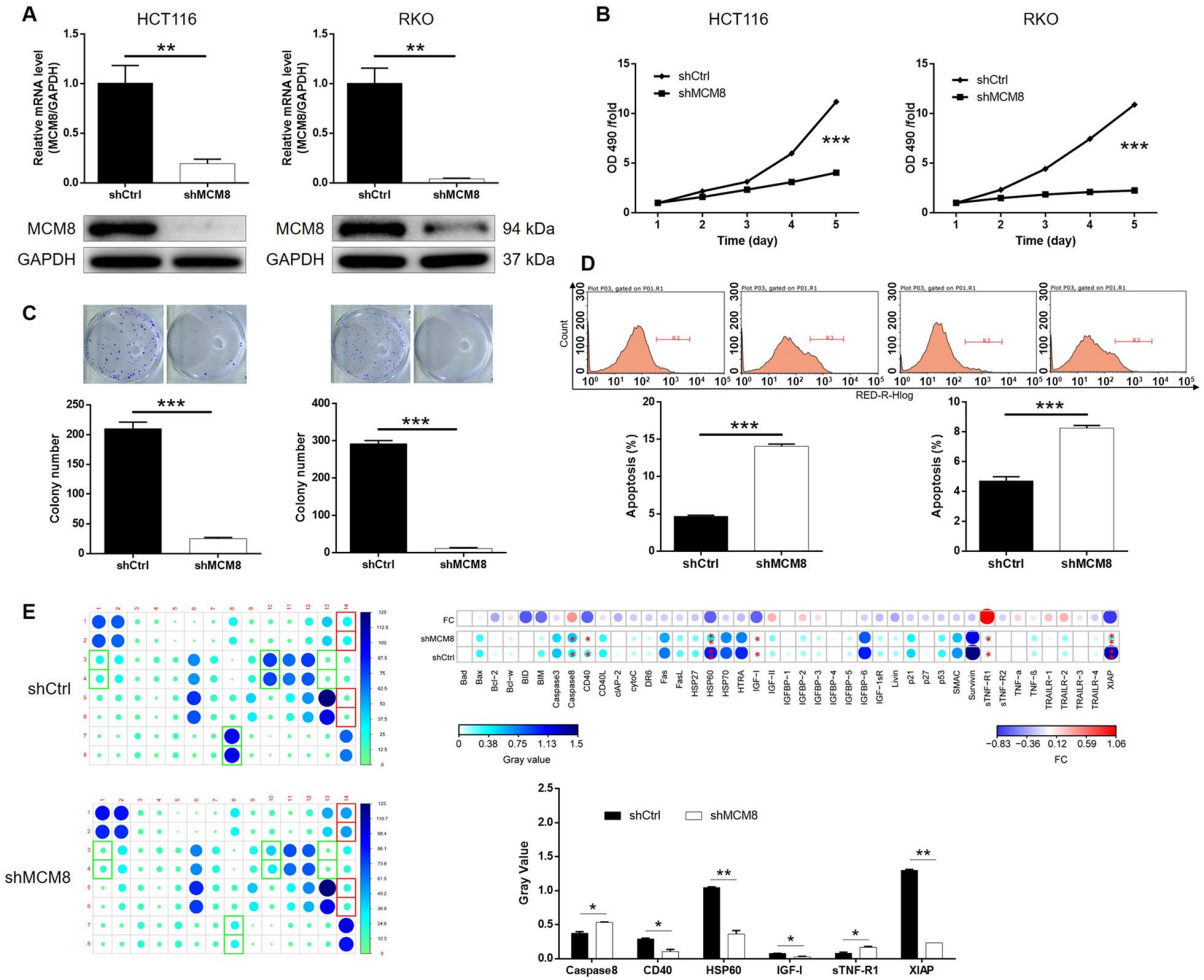
Knockdown of MCM8 inhibited development of CRCin vitro. **A** qPCR and western blotting were performed to detect the mRNA and protein expression of MCM8 in HCT116 and RKO cells transfected with shMCM8 or shCtrl. **B** MTT assay was utilized to determine cell proliferation of HCT116 and RKO cells transfected with shMCM8 or shCtrl. **C** Colony formation assay was used to evaluate the effects of MCM8 on cell growth of HCT116 and RKO cells. **D** Flow cytometry was performed to assess cell apoptosis of HCT116 and RKO cells transfected with shMCM8 or shCtrl. **E** A human apoptosis antibody array was used to recognize differential expressed proteins between shMCM8 and shCtrl groups of RKO cells. Representative images were randomly selected from 3 independent experiments. Data were shown as mean ± standard deviation (SD). *P<0.05, **P<0.01, ***P<0.001

For interfering endogenous MCM8 expression in CRC cells, shRNA targeting MCM8 was prepared and delivered into cells through lentivirus plasmids labelled with green fluorescent protein (GFP). According to the observation of fluorescence imaging, at least 80% cell population was transfected with lentivirus expressing shCtrl or shMCM8 (Additional file 1: Fig. S1A). The most efficient shMCM8 (RNAi-3) was recognized by qPCR and used in following experiments for silencing MCM8 (Additional file 1: Fig. S1B). The significantly downregulated mRNA and protein expression levels of MCM8 revealed by qPCR and western blotting delineated the successful construction of MCM8-interfereing cell models (Fig. 2A). Significantly suppressed reproductive capacity of HCT116 and RKO cells was observed after the knockdown of MCM8 (Fig. 2B). Consistently, the number of colonies formed in the same period by HCT116 and RKO cells was distinctly decreased in the absence of MCM8 (Fig. 2C). In contrast, ratio of apoptotic cells was markedly increased in CRC cells with MCM8 knockdown (Fig. 2D). Moreover, we performed an array analysis to distinguish the differential expression of apoptosis related proteins between cells with or without MCM8 knockdown, displaying the upregulation of Caspase8, sTNF-R1, and downregulation of CD40, HSP60, IGF-I and XIAP in shMCM8 cells (Fig. 2E). The in vitro studies showed MCM8 knockdown has a significant inhibitory impact on CRC development .

### Identification of CHSY1 as potential target of MCM8

**Fig. 3:**
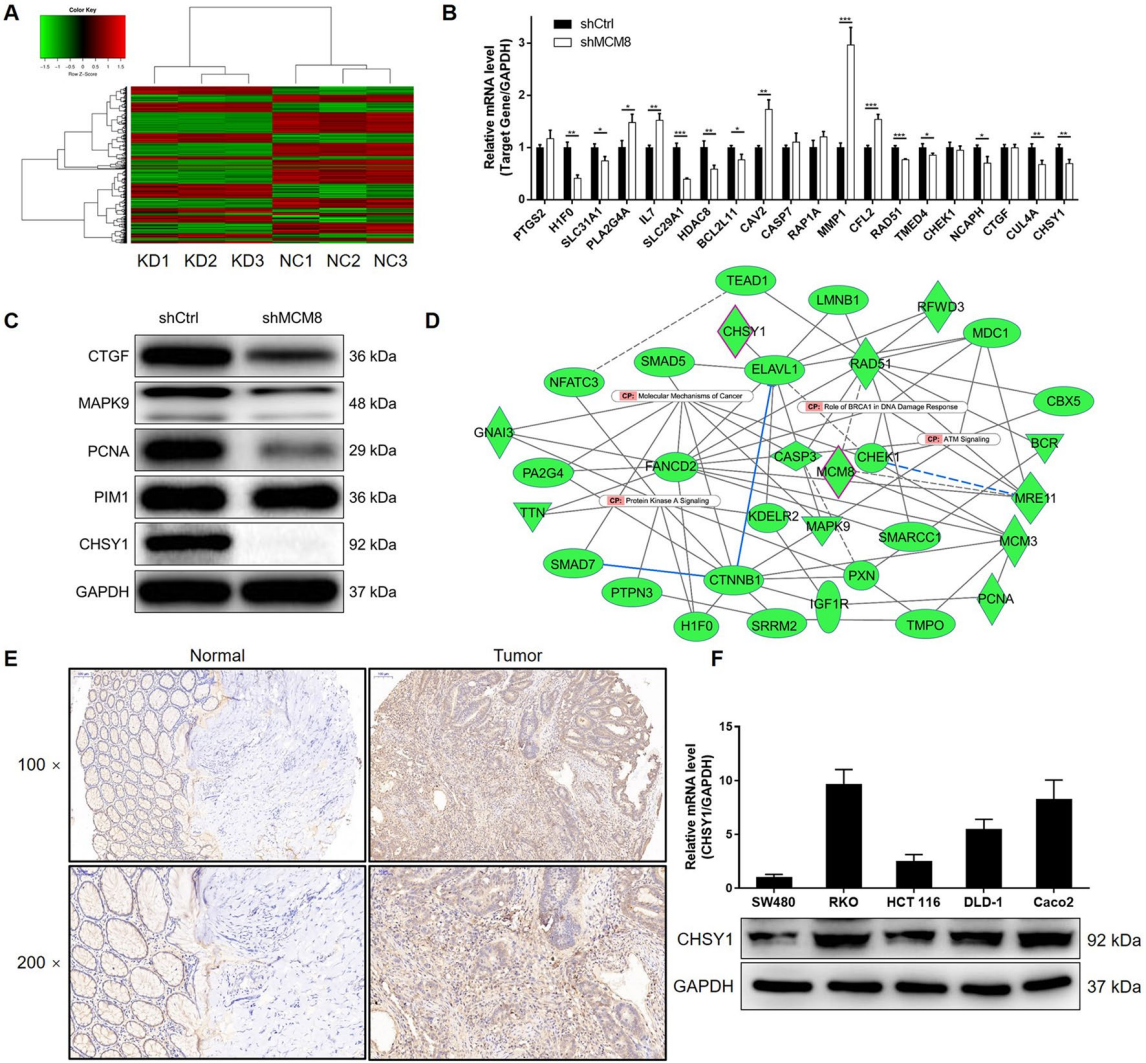
The exploration of target of MCM8 by RNA screening. **A** The heatmap was drawn based on the results of RNA sequencing performed on RKO cells with or without MCM8 knockdown (3 v 3). **B**, **C** Various candidates with downregulated expression in MCM8 silenced RKO cells were subjected to expression examination by qPCR (B) and western blotting (**C**). **D** MCM8 related interaction network was constructed based on IPA analysis and showed the potential linkage between MCM8 and CHSY1. **E** The expression of CHSY1 in CRC and normal tissues was detected by IHC. **F** The endogenous mRNA and protein expression of CHSY1 in CRC cell lines including HCT116, RKO, SW480, DLD-1 and Caco2 was examined by qPCR. Data were shown as mean ± standard deviation (SD). *P < 0.05, **P < 0.01, ***P < 0.001

In order to further explore the underlying mechanism of MCM8 in CRC, RNA sequencing was performed to screen differentially expressed genes (DEGs) induced by MCM8 knockdown in RKO cells (Fig. 3A, Additional file 1: Fig. S2A and S2B). Among the 524 DEGs, 220 of them was upregulated and the rest was downregulated. Based on the consideration of seeking similar tumor promotor as MCM8, several downregulated DEGs were selected as candidates, whose expression was detected in RKO cells. As shown in Fig. 3B, C, CHSY1 was found to show decreased mRNA and protein levels in RKO cells with MCM8 knockdown. Moreover, based on the enrichment of DEGs in canonical signalling pathways and IPA disease and function (Additional file 1: Fig. S2C and S2D), the involvement of CHSY1 in the significantly enriched ‘role of BRCA1 in DNA damage response’ (Additional file 1: Fig. S3) and CHSY1-related interaction network were assessed through IPA analysis (Fig. 3D), further verifying its potential as target of MCM8. As expected, the protein level of CHSY1 in tissues displayed similar trend with MCM8, namely upregulated expression in CRC (Fig. 3E). Abundant expression of CHSY1 in CRC cell lines was also revealed by the endogenous expression detection (Fig. 3F).

### MCM8 may regulate the expression of CHSY1 through affecting its ubiquitination

**Fig. 4:**
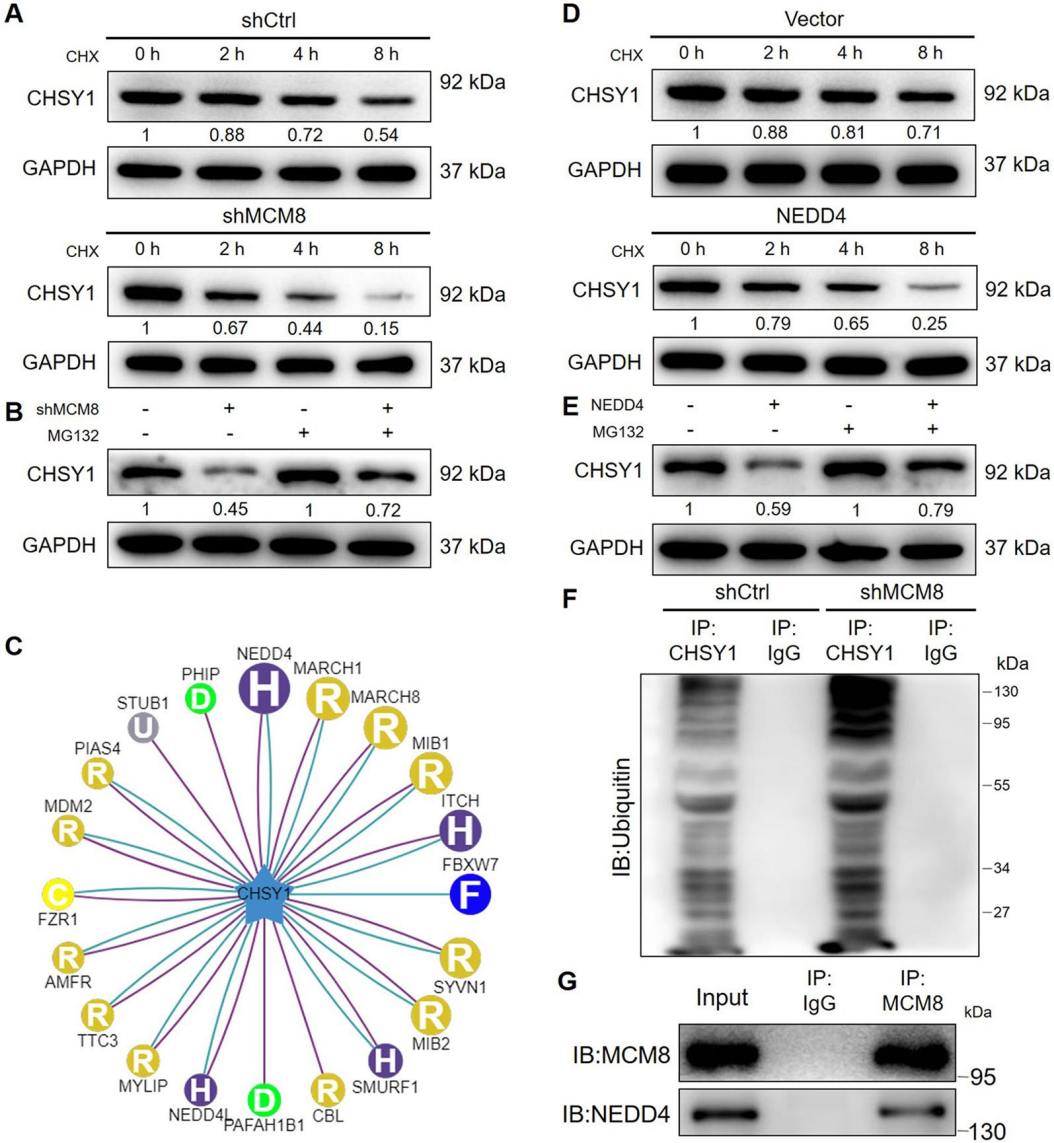
MCM8 may regulate the expression of CHSY1 through affecting its ubiquitination. **A** After the transfection with shCtrl or shMCM8, cells were treated with 100 µg/ml cycloheximide (CHX) followed by the western blot analysis of CHSY1 level at indicated time point. **B** The regulation of CHSY1 protein level by MCM8 knockdown was investigated with or without the treatment of MG132 (10 µM). **C** Online toolkit Ubibrowser was used for predicting potential E3 ubiquitin ligase of CHSY1. **D** After the transfection with Vector or NEDD4 (overexpression), cells were treated with 100 µg/ml cycloheximide (CHX) followed by the western blot analysis of CHSY1 level at indicated time point. **E** The regulation of CHSY1 protein level by NEDD4 overexpression was investigated with or without the treatment of MG132 (10 µM). **F** After the transfection with shCtrl or shMCM8, cells were harvested and cell lysates were subjected to immunoprecipitation with anti-CHSY1 antibody, using normal mouse IgG (IgG) as a control, followed by the western blot analysis with ubiquitin antibody. **G** Cell lysates were subjected to immunoprecipitation with anti-MCM8 antibody, using normal mouse IgG (IgG) as a control, followed by the western blot analysis with anti-MCM8 and anti-NEDD4 antibodies

For exploring the mechanism underlying the MCM8-induced regulation of CHSY1, we carried out a series of experiments on both RNA (transcription) level and protein level. As shown in Fig. 4A, a CHX-chase experiment showed that the degradation of CHSY1 protein in shMCM8 group is distinctly more rapid than that in shCtrl group. In other words, the protein stability of CHSY1 is relatively lower in the condition of MCM8 knockdown. Moreover, the influence on CHSY1 protein level by MCM8 knockdown could be abolished to some extent upon the treatment of MG132, a proteasome inhibitor, indicting the involvement of ubiquitin-proteasome system (UPS) (Fig. 4B). Accordingly, we consulted Ubibrowser (http://ubibrowser.ncpsb.org.cn/ubibrowser/strict/networkview/networkview/name/Q86X52) for predicting the E3 ubiquitin ligase of CHSY1, which indicated NEDD4 as the most promising candidate (Fig. 4C). Indeed, experimental verification displayed that NEDD4 overexpression also could decrease protein stability of CHSY1 under the mediation of UPS (Fig. 4D and E). The detection of ubiquitination level of CHSY1 further proved that MCM8 knockdown decreases CHSY1 protein stability as well as protein level through enhancing its ubiquitination (Fig. 4F). Moreover, the interaction between MCM8 and NEDD4, visualized by a co-immunoprecipitation assay, provides a possibility reason how MCM8 regulates the ubiquitination of CHSY1 (Fig. 4G).

### MCM8 and CHSY1 synergistically regulate CRC development in vitro

**Fig. 5:**
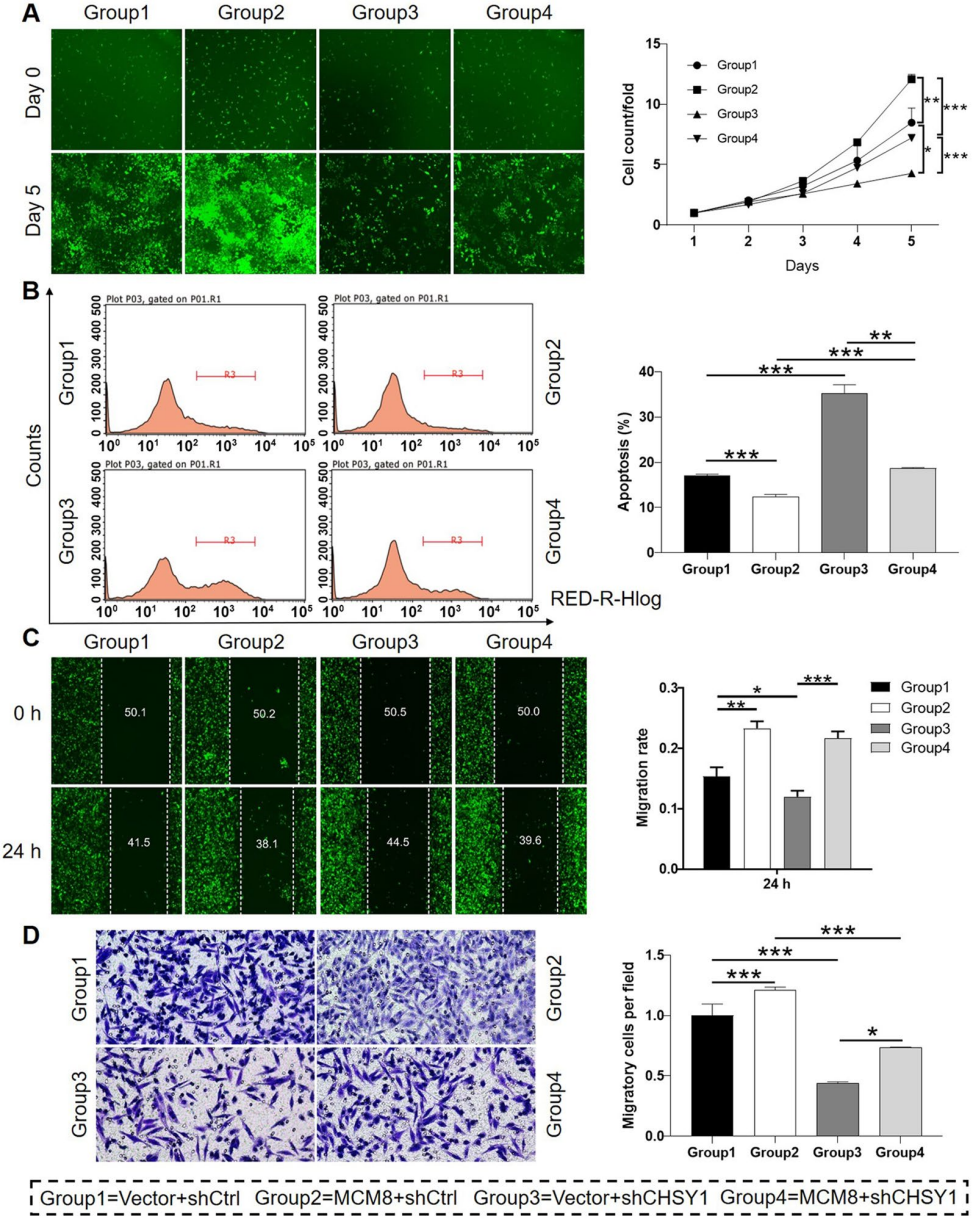
Knockdown of CHSY1 prohibits the impacts of MCM8 overexpression on CRC. **A** MTT assay was performed to investigate the effects of mere MCM8 overexpression (group 2), mere CHSY1 knockdown (group 3) or simultaneous MCM8 overexpression and CHSY1 knockdown (group 4) on cell proliferation. **B** Flow cytometry was utilized to show the effects of mere MCM8 overexpression (group 2), mere CHSY1 knockdown (group 3) or simultaneous MCM8 overexpression and CHSY1 knockdown (group 4) on cell apoptosis. **C**, **D** The effects of mere MCM8 overexpression (group 2), mere CHSY1 knockdown (group 3) or simultaneous MCM8 overexpression and CHSY1 knockdown (group 4) on cell migration were evaluated by wound-healing assay (**C**) and Transwell assay (**D**). Data were shown as mean ± standard deviation (SD). *P < 0.05, **P < 0.01, ***P < 0.001

To investigate whether CHSY1 exhibited functional impacts on CRC, RKO cells were transfected with lentivirus expressing the most efficient shCHSY1 for establishing CHSY1 knockdown model (Additional file 1: Fig. S4). With downregulated expression of CHSY1 relative to shCtrl group (Additional file 1: Fig. S5A and S5B), RKO cells in shCHSY1 group showed significantly lower proliferative activity, weaker cell migration ability and stronger spontaneous apoptosis (Additional file 1: Fig. S5C-S5F). Further taking the cells transfected with concurrent shMCM8 and shCHSY1 into account, it was observable that all the influence on the biological behaviours of RKO cells induced by CHSY1 could be aggravated by MCM8 knockdown (Additional file 1: Fig. S5). Furthermore, a ‘rescue’ functional experiment was further conducted to illustrate the dependence of MCM8-induced promotion of CRC on CHSY1. As shown in Fig. 5, it was demonstrated that overexpression of MCM8 exhibited strong promotion effects on CRC development through accelerating cell proliferation (Fig. 5A), inhibiting cell apoptosis (Fig. 5B) and enhancing cell mobility (Fig. 5C and D), which could be partially prohibited or even reversed in CHSY1-deficiency cells. Altogether, these results shed light on the role as tumor promotor of CHSY1 which may possess synergistic effects with MCM8 on CRC.

### MCM8 depletion impaired tumorigenesis of RKO cells in vivo

**Fig. 6:**
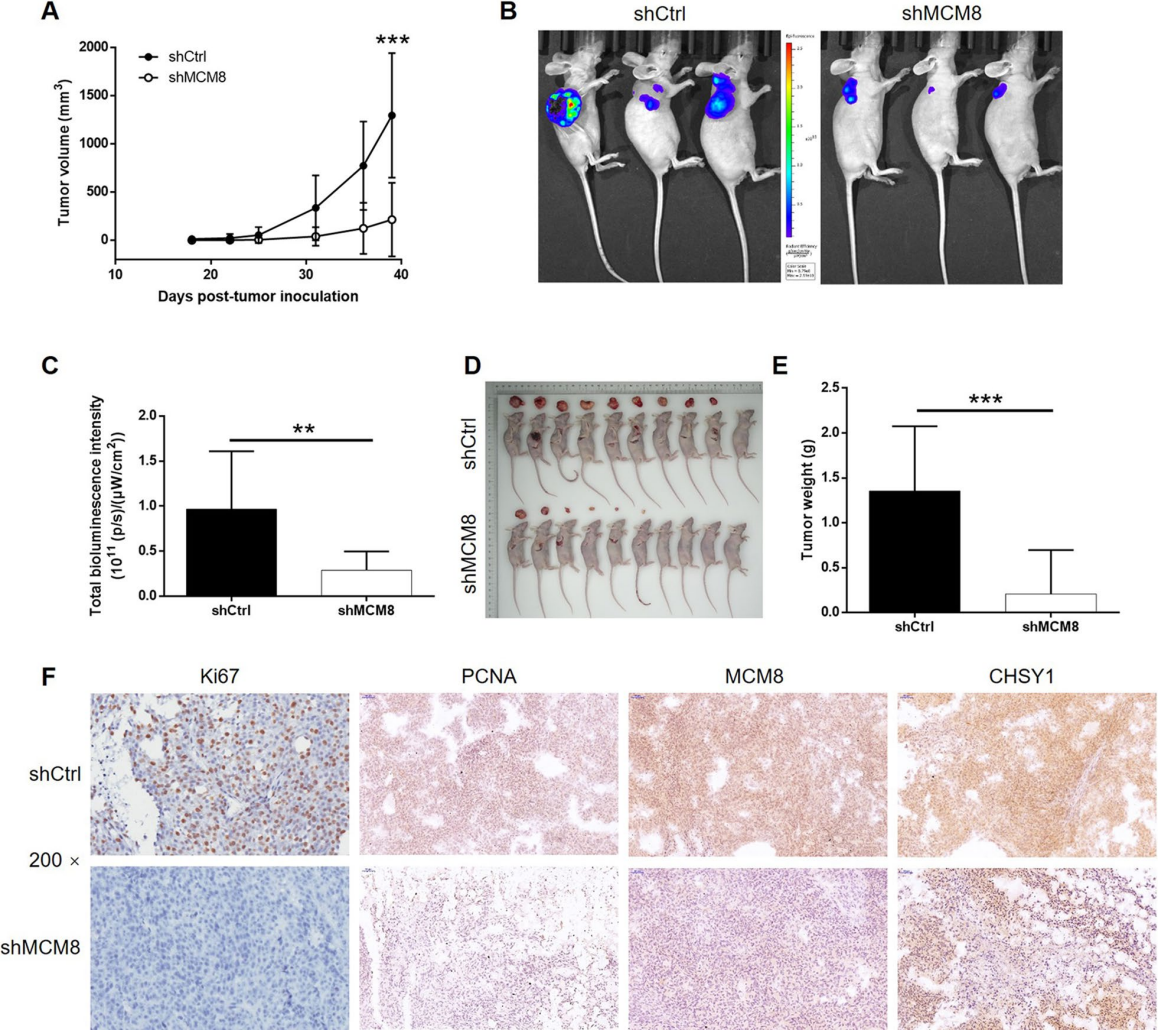
Knockdown of MCM8 inhibited growth of CRC in vivo. **A** From day 8 post injection of cells, the measurement of tumor volume was performed at indicated time intervals. **B** Bioluminescence imaging was performed to visualize the tumor in vivo, and the intensity of bioluminescence was scanned and used as a representation of tumor burden (**C**). **D**, **E** After sacrificing the mice, tumors were removed and collected for obtaining photos (**D**) and weighting (**E**). **F** Sections of xenografts were subjected to IHC analysis for detecting Ki-67, PCNA, MCM8 and CHSY1 expression. Data were shown as mean ± standard deviation (SD). *P < 0.05, **P < 0.01, ***P < 0.001

Despite of the clear role of MCM8 in CRC revealed by above results, its functions in vivo still needed to be verified. Therefore, the modified RKO cells were subcutaneously implanted into mice to demonstrate the effects of MCM8 knockdown on tumor growth. The continuous measurement of tumor volume showed remarkably slower grow rate of tumors formed by RKO cells with MCM8 knockdown relative to that formed by RKO cells transfected with shCtrl (Fig. 6A). In vivo imaging facilitated by bioluminescence of luciferase also validated much lighter tumor burden in mice implanted with MCM8 silenced RKO cells (Fig. 6B, C). After sacrificing the mice, xenografts were collected and weighted, showing similar results with the above examinations (Fig. 6D and E). Moreover, the sections of xenografts were subjected to IHC analysis for evaluating expression of Ki-67 and PCNA as a representation of tumor growth, indicating lower proliferation activity of xenograft removed from mice implanted with MCM8 silenced RKO cells (Fig. 6F). Finally, the levels of MCM8 and CHSY1 in xenografts were also verified by IHC (Fig. 6F). Collectively, we uncovered the suppression of CRC development by MCM8 knockdown in vivo.

## Discussion

The key finding in this study is the regulatory role of MCM8 in the development and progression of CRC. Although MCM8 used to be reported as a downregulated protein in CRC [26], the significantly increased protein expression of MCM8 in clinical CRC tissue samples relative to normal ones could be obviously observed and indicated the potential promotion effects of MCM8 on CRC in this study. The cellular functional detections based on cell models with silenced or ectopically expressed MCM8 also revealed that MCM8 may be capable of promoting CRC progression through facilitating cell proliferation, colony formation, cell movement, while disrupting cell apoptosis. Meanwhile, depletion of MCM8 also showed powerful ability in reducing the tumorigenicity of CRC cells.

MCM8 is a member of MCM family which, together with MCM9, attracted considerable attention recently. The investigation of Domenico et al. indicated that MCM8 acts as a helicase during the elongation phase of DNA replication, promotes the recruitment of RPA34, and stimulates the processing of DNA polymerase in the replication foci [27]. A mass number of evidences has suggested that MCM8 could form a complex with MCM9, thus playing vital role in DNA repairing and recombination. Lee et al. reported that the ATPase motifs of MCM8-MCM9 complex mediated the ensemble of MRE11 to foci of DNA damage, thus facilitating DNA resection regulated by MRE11-RAD50-NBS1 (MRN) at double-strand breaks. Moreover, the function diminishment experiments identified the potential role of MCM8-MCM9 complex in homologous recombination mediated repair of double-strand breaks in human cancers [17]. In addition, studies have clarified that knockout of MCM9 or knockdown of MCM8 can selectively sensitize transformed cells to cisplatin and olaparib. Mechanism studies showed that MCM8 knockdown may increase cell sensitivity to cisplatin or olaparib by increasing oncogene-induced replication stress [28]. In contrast to the relatively clear function of MCM8-MCM9 complex, the biological role of mere MCM8 especially in human cancers is still rarely investigated. As an example, Li and Ren et al. reported the key role of MCM8 in osteosarcoma through regulating cell phenotypes and in vivo tumor growth [22]. Bearing these in mind, this study fills in the gap of MCM8 function in the progression of CRC.

Through utilizing a human apoptosis antibody array, we identified the upregulation of Caspase8 and sTNF-R1, and downregulation of CD40, HSP60, IGF-I and XIAP in MCM8 knockdown cells, by which may MCM8 regulate the biological behaviours of CRC cells especially cell apoptosis. As a member of Caspase family, which possesses critical role in cell apoptosis, Caspase8 has been demonstrated to act as regulator of cell apoptosis thus mediating the regulation of CRC development by various molecules [29, 30]. CD40, together with its ligand CD40L, has also been reported to be involved in various functions such as cell proliferation and cell apoptosis in CRC [31]. Recently, HSP60 was found to be a potential target of UBXN2A-CHIP axis in the regulation of CRC by the studies of Sane et al. [32]. Li et al. identified a feedback loop between IGF-I and microRNA let-7e, which was capable of modulating proliferation and migration of CRC cells [33]. Besides, previous work has not only indicated the oncoprotein-like properties of XIAP in cancer, but also revealed its functions in mediating regulation of cell apoptosis in cancer [34].

In our study, the attempt to explore the underlying mechanism as well as downstream target of MCM8 was made through a genechip. Among the variety of differentially expressed genes, CHSY1 which was significantly downregulated in MCM8 deficiency cells, involved in the significantly enriched signalling pathway and was found to possess potential interaction with MCM8 by IPA analysis, attracted our attention.

Chondroitin sulfate (CS) is widely distributed on the extracellular matrix and cell membrane surface of various tissues [35]. It plays an important role in the development of brain neural networks, inflammatory response, infection, cell division and tissue morphology [36]. At the same time, it possesses physiological functions such as inhibiting axonal regeneration after spinal cord injury [37], preventing abnormal myocardial remodeling [38] and so on. Moreover, studies have revealed the capability of CS in the regulation of malignant tumors. For example, it was reported that shark CS could inhibit liver cancer, induce apoptosis of multiple myeloma and breast cancer cells, and slow down the growth of tumors in mice bearing breast cancer [39]. CHPF is one of the six essential glycosyltransferases in the biosynthesis of CS, which acts as necessary auxiliary factor during the synthesis of repetitive disaccharide unit in CS [35]. Considering the physiological functions of CS, it was supposed that CHSY1 may regulate cell division and differentiation, thus participating in the regulation of body development and disease occurrence. Moreover, recent studies have revealed the role of CHSY1 in several types of malignant tumors. For example, Momose et al. reported that CHSY1 expression was obviously higher in myxofibrosarcoma and malignant peripheral nerve sheath tumor compared with other tumors and significantly associated malignant grade, whose high expression predicted poor prognosis [40]. CHSY1 was also found to be able to regulate hedgehog signaling, thus promoting the malignant behaviors of cancer cells of hepatocellular carcinoma [41]. Notably, the work of Zeng et al. suggested that CHSY1 could promote cell proliferation of colorectal cancer through regulating NF-κB and Caspase-3/7 signaling pathway [42]. Our results also confirmed that CHSY1 was upregulated in CRC tissues and abundantly expressed in CRC cells. Functionally, CHSY1 knockdown could further aggregate the inhibition effects of MCM8 interference on CRC, while alleviate the promotion effects of MCM8 overexpression on CRC. Mechanistically, it was illustrated that MCM8 may regulate CHSY1 expression through influencing its NEDD4-mediated ubiquitination and thus the UPS.

In conclusion, MCM8 was identified as a tumor promotor of CRC, which is upregulated in CRC tissues compared with normal tissues and capable of promoting CRC progression through regulating cell proliferation, cell apoptosis and cell migration. Moreover, CHSY1 was recognized as a potential target of MCM8, which could regulate expression of each other and synergistically promote CRC. Collectively, MCM8 may be a promising therapeutic target and prognostic indicator of CRC in the future.

### Supplementary Information

**Additional file 1:** **Figure S1.** (A) The transfection efficiencies of shMCM8 andshCtrl in HCT116 and RKO cells were evaluated through observing thefluorescence of GFP on lentivirus vector. (B) The ability of 3 shRNAs tosilence MCM8 was evaluated by qPCR. Data were shown as mean ± standarddeviation (SD). *P<0.05, **P<0.01, ***P<0.001. **Figure S2.** (A) The scatter plot of gene expressionprofiling in RKO cells with or without MCM8 knockdown. (B) The volcano plot ofgene expression profiling in RKO cells with or without MCM8 knockdown. Red dotsrepresent the DEGs. (C) The enrichment of the DEGs in canonical signalingpathways was analyzed by IPA. (D) The enrichment of the DEGs in IPA disease andfunction was analyzed by IPA. **Figure S3.** The histogram of Role of BRCA1 in DNA damage response pathway. **Figure S4.** (A) The transfectionefficiencies of shCtrl, shCHSY1, shMCM8+shCHSY1 in RKO cells were evaluatedthrough observing the fluorescence of GFP on lentivirus vector. (B) Theknockdown efficiencies of 3 shRNAs designed for CHSY1 knockdown were evaluatedby qPCR. Data was shown as mean ± SD. **P< 0.01. **Figure S5.** Knockdown ofCHSY1 deepens the impacts of MCM8 depletion on CRC. (A) The mRNA expression ofCHSY1 and MCM8 in different groups of cells was detected by qPCR. (B) Theprotein levels of CHSY1 and MCM8 in different groups of cells were detected bywestern blotting. (C) MTT assay was performed to investigate the effects ofCHSY1 knockdown or simultaneous CHSY1 and MCM8 knockdown on cell proliferation.(D) Flow cytometry was utilized to show the effects of CHSY1 knockdown orsimultaneous CHSY1 and MCM8 knockdown on cell apoptosis. (E, F) The effects ofCHSY1 knockdown or simultaneous CHSY1 and MCM8 knockdown on cell migration wereevaluated by wound-healing assay (E) and Transwell assay (F). Representativeimages were randomly selected from 3 independent experiments. Data were shownas mean ± standard deviation (SD). *P<0.05,**P<0.01, ***P<0.001. **Table S1.** Antibodies used in western blotting and IHC. **Table S2.** The target sequences andshRNA sequences. **Table S3.** Primersused in qPCR. **Table S4.** Relationshipbetween MCM8 expression and tumor characteristics in patients with colorectalcancer analyzed by Spearman rank correlation analysis.

## Data Availability

The data generated in this study are available within the article and its supplementary data files.

## Abbreviations

CRC: Colorectal cancer
MCM: Microchromosome maintenance complex
ceRNA: Competing endogenous RNA
IHC: Immunohistochemical
ECL: Enhanced chemiluminescence
SD: Standard deviation
GFP: Green fluorescent protein
DEGs: Differentially expressed genes
MRN: MRE11-RAD50-NBS1
