# Supplementary material for: Function and mechanism of MCM8 in the development and progression of colorectal cancer
Source: J Transl Med. 2023 Sep 14;21:623. doi: 10.1186/s12967-023-04084-9 (PMC10503009; doi:10.1186/s12967-023-04084-9)
Supplement: Supplementary file 1 — Additional file 1: Figure S1. (A) The transfection efficiencies of shMCM8 andshCtrl in HCT116 and RKO cells were evaluated through observing thefluorescence of GFP on lentivirus vector. (B) The ability of 3 shRNAs tosilence MCM8 was evaluated by qPCR. Data were shown as mean ± standarddeviation (SD). *P<0.05, **P<0.01, ***P<0.001. Figure S2. (A) The scatter plot of gene expressionprofiling in RKO cells with or without MCM8 knockdown. (B) The volcano plot ofgene expression profiling in RKO cells with or without MCM8 knockdown. Red dotsrepresent the DEGs. (C) The enrichment of the DEGs in canonical signalingpathways was analyzed by IPA. (D) The enrichment of the DEGs in IPA disease andfunction was analyzed by IPA. Figure S3. The histogram of Role of BRCA1 in DNA damage response pathway. Figure S4. (A) The transfectionefficiencies of shCtrl, shCHSY1, shMCM8+shCHSY1 in RKO cells were evaluatedthrough observing the fluorescence of GFP on lentivirus vector. (B) Theknockdown efficiencies of 3 shRNAs designed for CHSY1 knockdown were evaluatedby qPCR. Data was shown as mean ± SD. **P< 0.01. Figure S5. Knockdown ofCHSY1 deepens the impacts of MCM8 depletion on CRC. (A) The mRNA expression ofCHSY1 and MCM8 in different groups of cells was detected by qPCR. (B) Theprotein levels of CHSY1 and MCM8 in different groups of cells were detected bywestern blotting. (C) MTT assay was performed to investigate the effects ofCHSY1 knockdown or simultaneous CHSY1 and MCM8 knockdown on cell proliferation.(D) Flow cytometry was utilized to show the effects of CHSY1 knockdown orsimultaneous CHSY1 and MCM8 knockdown on cell apoptosis. (E, F) The effects ofCHSY1 knockdown or simultaneous CHSY1 and MCM8 knockdown on cell migration wereevaluated by wound-healing assay (E) and Transwell assay (F). Representativeimages were randomly selected from 3 independent experiments. Data were shownas mean ± standard deviation (SD). *P<0.05,**P<0.01, ***P<0.001. Table S1. Antibodies used in western blotting [file 12967_2023_4084_MOESM1_ESM.docx]

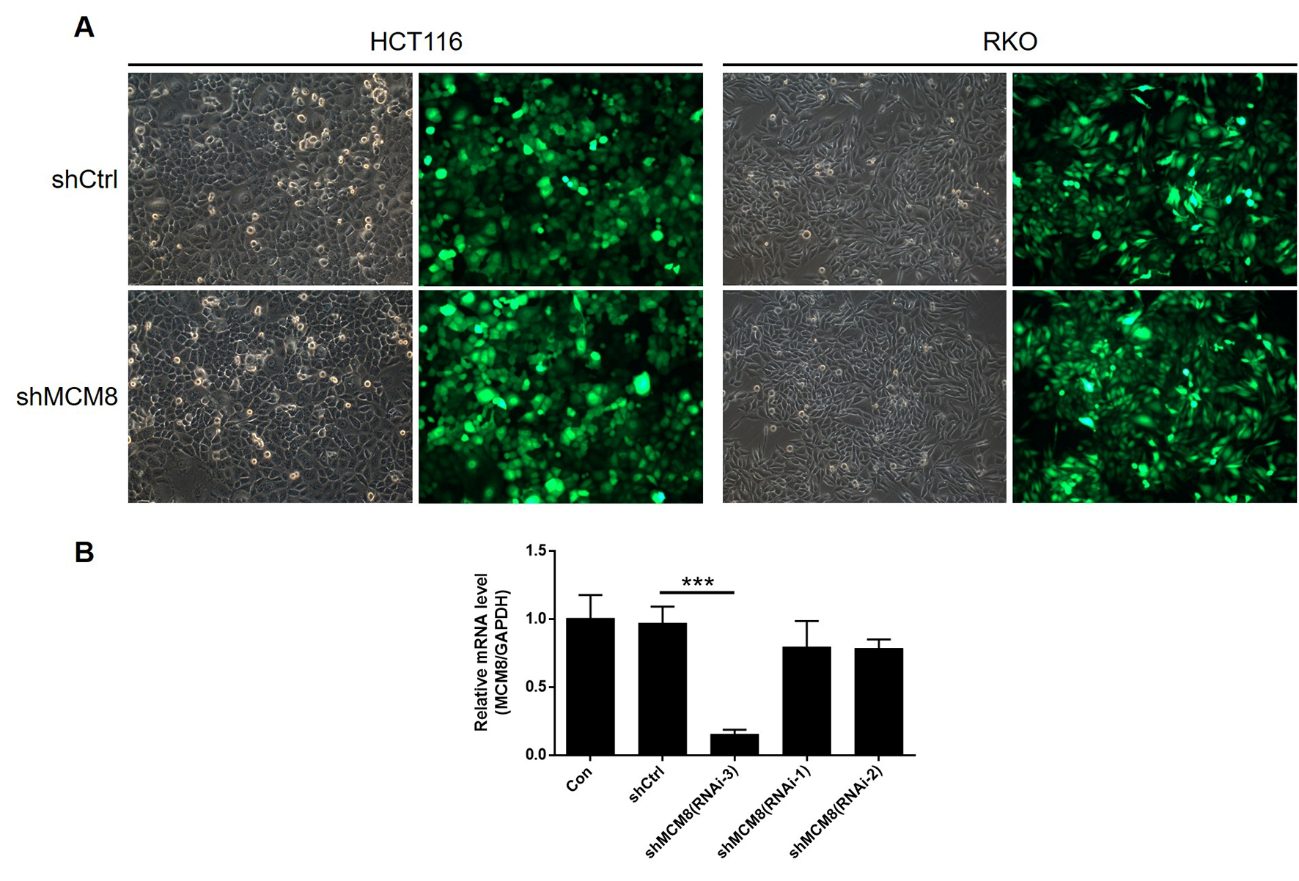


**Figure S1.** (A) The transfection efficiencies of shMCM8 and shCtrl in HCT116 and RKO cells were evaluated through observing the fluorescence of GFP on lentivirus vector. (B) The ability of 3 shRNAs to silence MCM8 was evaluated by qPCR. Data were shown as mean ± standard deviation (SD). **P*<0.05, ***P*<0.01, ****P*<0.001


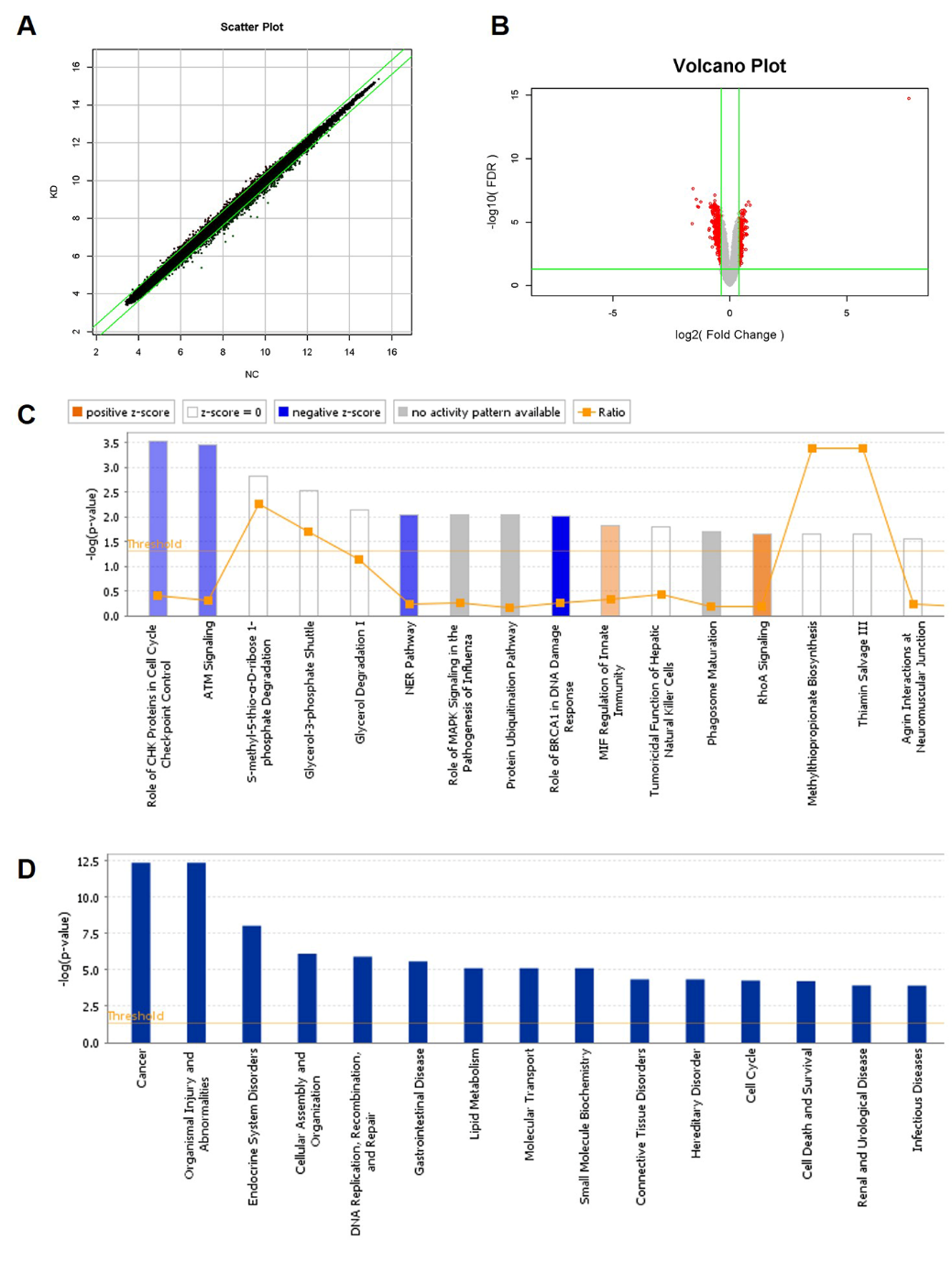


**Figure S2.** (A) The scatter plot of gene expression profiling in RKO cells with or without MCM8 knockdown. (B) The volcano plot of gene expression profiling in RKO cells with or without MCM8 knockdown. Red dots represent the DEGs. (C) The enrichment of the DEGs in canonical signaling pathways was analyzed by IPA. (D) The enrichment of the DEGs in IPA disease and function was analyzed by IPA.


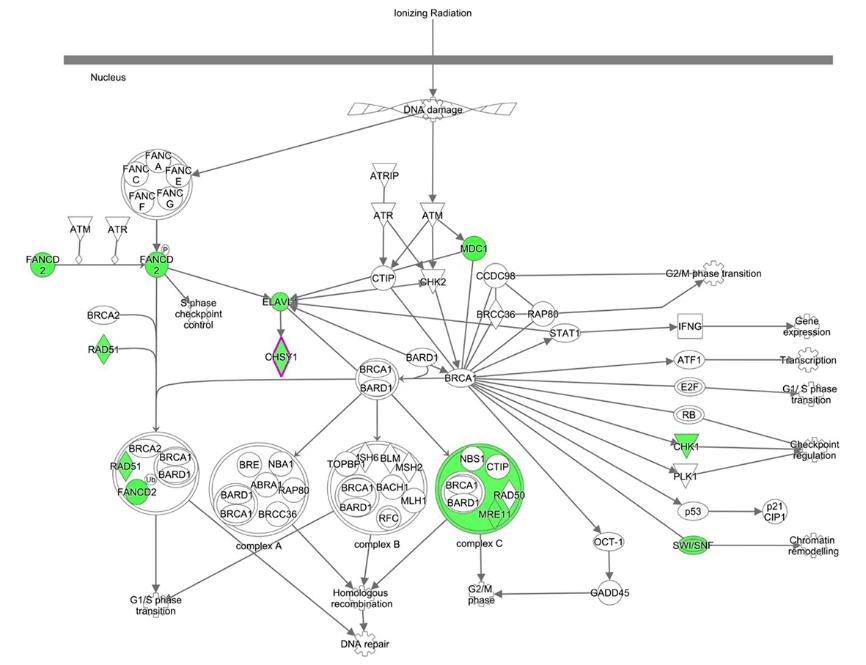


**Figure S3.** The histogram of Role of BRCA1 in DNA damage response pathway.


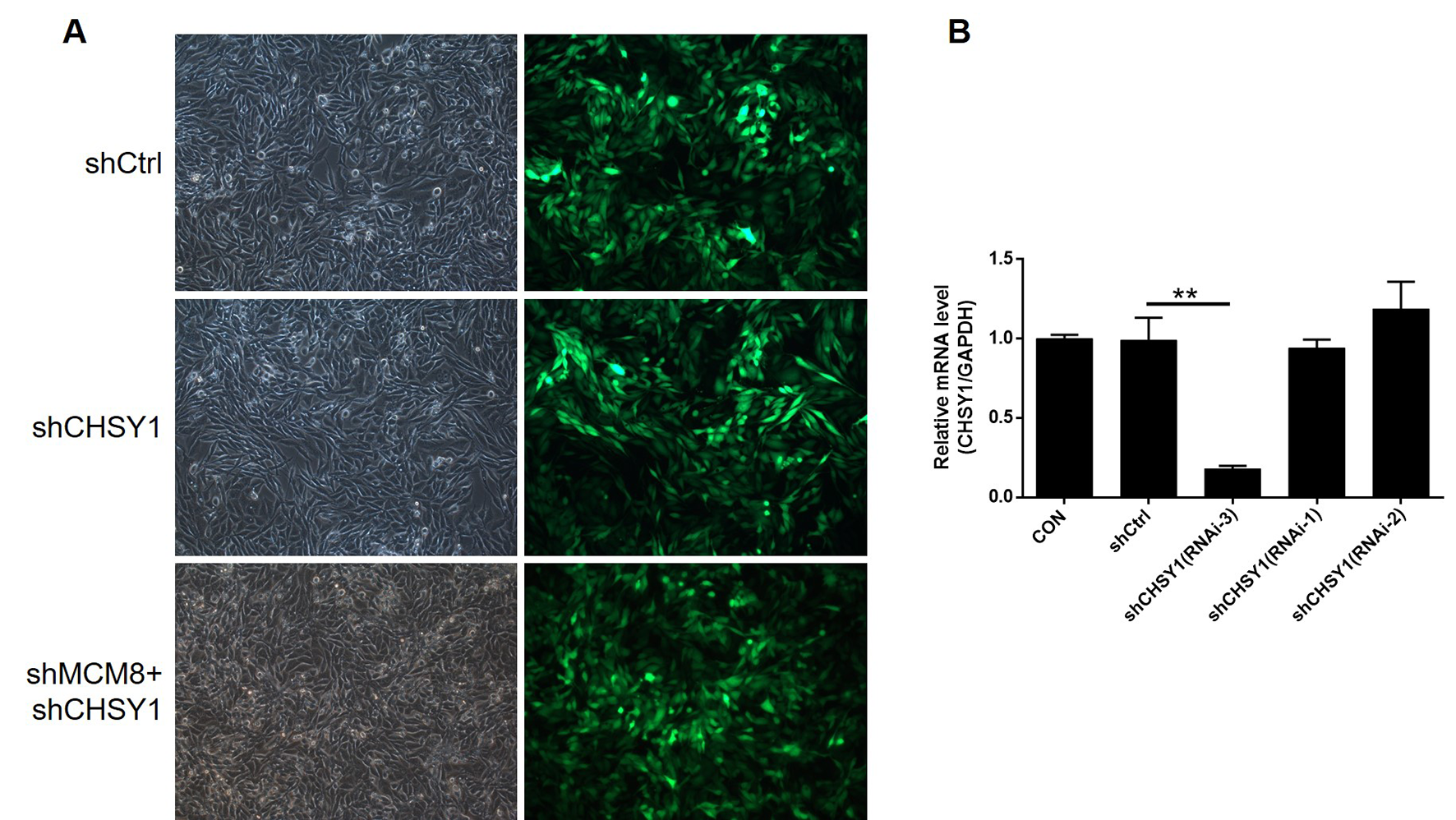


**Figure S4.** (A) The transfection efficiencies of shCtrl, shCHSY1, shMCM8+shCHSY1 in RKO cells were evaluated through observing the fluorescence of GFP on lentivirus vector. (B) The knockdown efficiencies of 3 shRNAs designed for CHSY1 knockdown were evaluated by qPCR. Data was shown as mean ± SD. ***P* < 0.01


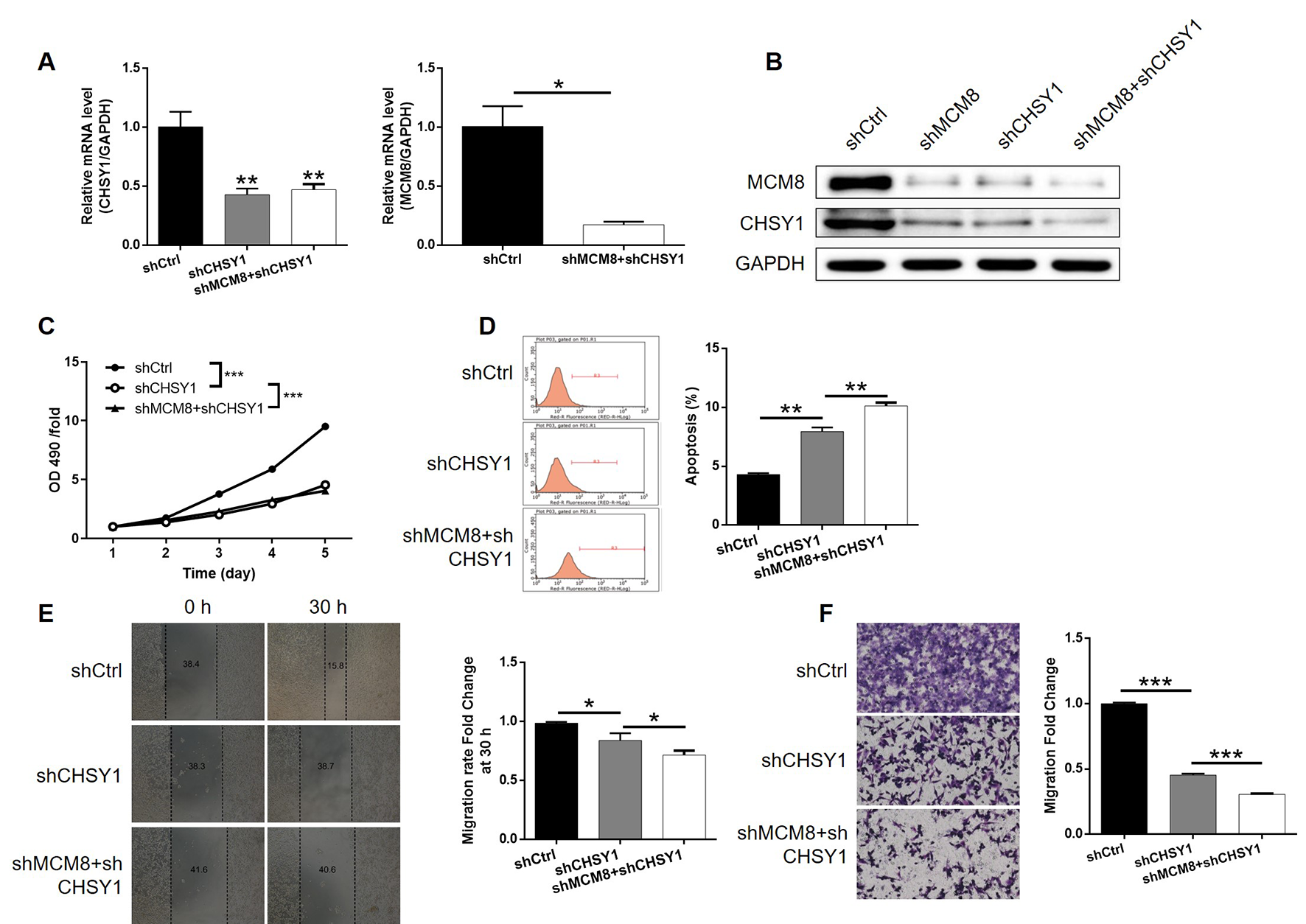


**Figure S5. Knockdown of CHSY1 deepens the impacts of MCM8 depletion on CRC.** (A) The mRNA expression of CHSY1 and MCM8 in different groups of cells was detected by qPCR. (B) The protein levels of CHSY1 and MCM8 in different groups of cells were detected by western blotting. (C) MTT assay was performed to investigate the effects of CHSY1 knockdown or simultaneous CHSY1 and MCM8 knockdown on cell proliferation. (D) Flow cytometry was utilized to show the effects of CHSY1 knockdown or simultaneous CHSY1 and MCM8 knockdown on cell apoptosis. (E, F) The effects of CHSY1 knockdown or simultaneous CHSY1 and MCM8 knockdown on cell migration were evaluated by wound-healing assay (E) and Transwell assay (F). Representative images were randomly selected from 3 independent experiments. Data were shown as mean ± standard deviation (SD). **P*<0.05, ***P*<0.01, ****P*<0.001

Table S1 Antibodies used in western blotting and IHC

| Primary antibodies | Dilution in WB | Source species | Company | Catalog No. |
| --- | --- | --- | --- | --- |
| MCM8 | 1:1000 | Rabbit | Invitrogen | PA5-41325 |
| GAPDH | 1:3000 | Rabbit | Bioworld | AP0063 |
| CTGF | 1:1000 | Rabbit | abcam | ab6992 |
| MAPK9 | 1:1000 | Rabbit | abcam | ab76125 |
| PCNA | 1:1000 | Mouse | abcam | ab29 |
| PIM1 | 1:1000 | Rabbit | abcam | ab98004 |
| CHSY1 | 1:500 | Rabbit | biorbyt | orb126811 |
| Ubiquitin | 1:1000 | Mouse | Santa Cruz | sc-47721 |
| CHSY1 (co-IP) | 1:50 | Rabbit | Santa Cruz | sc-292185 |
| NEDD4 | 1:500 | Rabbit | Proteintech | 21698-1-AP |
| Primary antibodies | Dilution in IHC | Source species | Company | Catalog No. |
| MCM8 | 1:200 | Rabbit | Thermo | PA5-41325 |
| CHSY1 | 1:100 | Rabbit | biorbyt | orb126811 |
| Ki67 | 1:200 | Rabbit | abcam | ab16667 |
| PCNA | 1:100 | Mouse | Abcam | Ab29 |
|  |  |  |  |  |
| Secondary antibody | Dilution |  | Company | Catalog No. |
| HRP Goat Anti-Rabbit IgG (WB) | 1:3000 |  | Beyotime | A0208 |
| HRP Goat Anti-Mouse IgG (WB) | 1:3000 |  | Beyotime | A0216 |
| HRP Goat Anti-Rabbit IgG (IHC) | 1:400 |  | abcam | ab97080 |
| HRP Goat Anti-Rabbit IgG (IHC) | 1:200 |  | Beyotime | 32430 |

Table S2 The target sequences and shRNA sequences

| Gene | No. | Target sequence (5'-3') | shRNA sequences (5'-3') |
| --- | --- | --- | --- |
| MCM8 | Pbr11106-a | TGGCAATACATCAGGTGTTAA | CcggTGGCAATACATCAGGTGTTAActcgagTTAACACCTGATGTATTGCCATTTTTg |
| MCM8 | Pbr11106-b | TGGCAATACATCAGGTGTTAA | aattcaaaaaTGGCAATACATCAGGTGTTAActcgagTTAACACCTGATGTATTGCCA |
| MCM8 | Pbr11107-a | CTGGAATTGTCAAAGTCTCAA | CcggCTGGAATTGTCAAAGTCTCAActcgagTTGAGACTTTGACAATTCCAGTTTTTg |
| MCM8 | Pbr11107-b | CTGGAATTGTCAAAGTCTCAA | aattcaaaaaCTGGAATTGTCAAAGTCTCAActcgagTTGAGACTTTGACAATTCCAG |
| MCM8 | Pbr11108-a | AGGCAGCTGGAATCTTTGATT | CcggAGGCAGCTGGAATCTTTGATTctcgagAATCAAAGATTCCAGCTGCCTTTTTTg |
| MCM8 | Pbr11108-b | AGGCAGCTGGAATCTTTGATT | aattcaaaaaAGGCAGCTGGAATCTTTGATTctcgagAATCAAAGATTCCAGCTGCCT |
| CHSY1 | Pbr14695-a | GCACAAAGAACCCAAAGATAA | CcggGCACAAAGAACCCAAAGATAActcgagTTATCTTTGGGTTCTTTGTGCTTTTTg |
| CHSY1 | Pbr14695-b | GCACAAAGAACCCAAAGATAA | aattcaaaaaGCACAAAGAACCCAAAGATAActcgagTTATCTTTGGGTTCTTTGTGC |
| CHSY1 | Pbr14696-a | TGAGAATTACGAGCAGAACAA | CcggTGAGAATTACGAGCAGAACAActcgagTTGTTCTGCTCGTAATTCTCATTTTTg |
| CHSY1 | Pbr14696-b | TGAGAATTACGAGCAGAACAA | aattcaaaaaTGAGAATTACGAGCAGAACAActcgagTTGTTCTGCTCGTAATTCTCA |
| CHSY1 | Pbr14697-a | GCAAATACAGCAACACAGAAA | CcggGCAAATACAGCAACACAGAAActcgagTTTCTGTGTTGCTGTATTTGCTTTTTg |
| CHSY1 | Pbr14697-b | GCAAATACAGCAACACAGAAA | aattcaaaaaGCAAATACAGCAACACAGAActcgagTTTCTGTGTTGCTGTATTTGC |
|  |  |  |  |

Table S3 Primers used in qPCR

| Gene | Forward primer sequence (5’-3’) | Reverse primer sequence (5’-3’) |
| --- | --- | --- |
| GAPDH | TGACTTCAACAGCGACACCCA | CACCCTGTTGCTGTAGCCAAA |
| MCM8 | TGAGTTACAAGCCCAGGAAGG | GCAGCACAAAGAAAAGCCATCT |
| PTGS2 | CAAATCCTTGCTGTTCCCACC | TTTCTCCATAGAATCCTGTCCG |
| H1F0 | CCACAGACCACCCCAAGTATT | CTTGATCTGCGAGTCAGCGT |
| SLC31A1 | GGAGCTTTTGTGGCAGTGTTT | GAATGCTGACTTGTGACTTACGC |
| PLA2G4A | TTGTGCTACCTACGTTGCTGG | AATCTCCTCTGGCCCTTTCTC |
| IL7 | GACTTCCTCCCCTGATCCTTGT | CGATGCTGACCATTAGAACACTC |
| SLC29A1 | CCCTGCTGTTATTCACCTACCTC | AAGAAGGGCAGAGCATCCAG |
| HDAC8 | GTGTAAATGTGCCCATTCAGGA | CCAATTCCCACTGGAGTCATG |
| BCL2L11 | TCCTGAAGGCAATCACGGAG | TGAAAAGCGGGGATCTGGTA |
| CAV2 | TCGAGGATGTGATCGCAGAG | GGAACACCGTCAGGAACTTGTA |
| CASP7 | GATTTGACAGCCCACTTTAGGG | ATCATCAAGCTCGGTCCCTC |
| RAP1A | ACGGGTTAAGGACACGGAA | TGCCAACTACTCGCTCATCTT |
| MMP1 | ACGATTCGGGGAGAAGTGATG | TGTCGGCAAATTCGTAAGCAG |
| CFL2 | TGTACGATGCCACATACGAAAC | CAAGCCATTTACTTGCCACTCA |
| RAD51 | CAGTGATGTCCTGGATAATGTAGC | TGTTCTGTAAAGGGCGGTGG |
| TMED4 | CCAGGGGCTCTACTTCCACA | ACGATAGTTGCCGATGACCA |
| CHEK1 | TTGGCTTGGCAACAGTATTTCG | CCAGCGAGCATTGCAGTAAGT |
| NCAPH | GATTACAACAACCCTAACGACACC | AGGTCAGAGTTCCCAACAGGTC |
| CTGF | GGTGTGGCTTTAGGAGCAGT | TGATGGCTGGAGAATGCACA |
| CUL4A | CAACAAAGAAGCCACAGACGA | CTGAGGCACTTTTCCCAACA |
| CHSY1 | AGTGGGTGGCTTTGATGTTTC | AGGATGGTGGACGTGGACTA |

Table S4 Relationship between MCM8 expression and tumor characteristics in patients with colorectal cancer analyzed by Spearman rank correlation analysis

| Tumor characteristics | index |  |
| --- | --- | --- |
| Grade | Spearman correlation | 0.243 |
|  | Significance (two tailed) | 0.016 |
|  | n | 98 |
